# Health Effects of Lesion Localization in Multiple Sclerosis: Spatial Registration and Confounding Adjustment

**DOI:** 10.1371/journal.pone.0107263

**Published:** 2014-09-18

**Authors:** Ani Eloyan, Haochang Shou, Russell T. Shinohara, Elizabeth M. Sweeney, Mary Beth Nebel, Jennifer L. Cuzzocreo, Peter A. Calabresi, Daniel S. Reich, Martin A. Lindquist, Ciprian M. Crainiceanu

**Affiliations:** 1 Department of Biostatistics, Bloomberg School of Public Health, Johns Hopkins University, Baltimore, Maryland, United States of America; 2 Department of Biostatistics and Epidemiology, Perelman School of Medicine, University of Pennsylvania, Philadelphia, Pennsylvania, United States of America; 3 Translational Neurology Unit, Neuroimmunology Branch, National Institute of Neurological Disorders and Stroke, National Institutes of Health, Bethesda, Maryland, United States of America; 4 Laboratory for Neurocognitive and Imaging Research, Kennedy Krieger Institute, Baltimore, Maryland, United States of America; 5 Department of Neurology, The Johns Hopkins University School of Medicine, Baltimore, Maryland, United States of America; 6 Department of Radiology, The Johns Hopkins University School of Medicine, Baltimore, Maryland, United States of America; University Medical Center Göttingen, Germany

## Abstract

Brain lesion localization in multiple sclerosis (MS) is thought to be associated with the type and severity of adverse health effects. However, several factors hinder statistical analyses of such associations using large MRI datasets: 1) spatial registration algorithms developed for healthy individuals may be less effective on diseased brains and lead to different spatial distributions of lesions; 2) interpretation of results requires the careful selection of confounders; and 3) most approaches have focused on voxel-wise regression approaches. In this paper, we evaluated the performance of five registration algorithms and observed that conclusions regarding lesion localization can vary substantially with the choice of registration algorithm. Methods for dealing with confounding factors due to differences in disease duration and local lesion volume are introduced. Voxel-wise regression is then extended by the introduction of a metric that measures the distance between a patient-specific lesion mask and the population prevalence map.

## Introduction

Multiple sclerosis (MS) is an inflammatory demyelinating disease that results in the formation of brain lesions that can cause various debilitating effects. The use of magnetic resonance imaging (MRI) has allowed researchers to investigate the occurrence patterns of these lesions. It has been shown that the nature of the resulting disabilities depends heavily on the location of lesions in the brain [1]. However, the problem is complicated, as increased disability may not correspond to noticeable changes in MRI scans or to a significant increase in the lesion burden in the patients. Alternatively, new lesions may form without resulting in a change in disability indices. Hence, there is a need for more detailed information about the association between brain lesion location and MS disease severity. In this study, we quantify MS disease severity using a common disability score: the Extended Disability Status Scale (EDSS, [2]).

The study of lesion localization has a successful history with several major quantitative results. In particular, [3] showed that there is an association between lesion load and disability score using standard linear regression at every voxel. Areas of significant association were estimated using thresholds of t-statistic maps. In later work, [4] investigated the association between average cortical thickness, lesion load and disability score, showing that cortical atrophy occurs even in MS patients with low disability scores. In a related work, [5] and [6] discussed the association of gray matter volume reduction with white matter lesion localization. [7] found a statistically significant association between lesion location and disease severity scores based on non-parametric permutation tests.

More recently, [8] described the spatiotemporal distribution of white matter lesions in patients with relapsing-remitting MS as compared with patients with secondary progressive MS. In addition, [9] investigated the effect of white matter lesion distribution on cognition in MS and found that in cognitively impaired patients, lesions are not as widespread as in cognitively preserved patients though the volume is higher in the first case. Finally, [10] commented on the association between normal cerebral perfusion patterns and white matter lesion distributions.

All these analyses depend heavily on the ability to compare the location of white matter lesions across subjects. The interpretations of inferences rely on a common template space and are, thus, intrinsically linked to the spatial registration algorithm implemented for preprocessing. The majority of these findings are obtained using affine registration (e.g. [11]), while the effect of the specific choice of registration algorithm has not been addressed. Additionally, registration algorithms have only been validated on healthy controls, while their performance on abnormal brains remains unknown. This may be a serious problem in MS, where brain images contain severe pathologies including enlarged cerebrospinal fluid spaces and lesions whose location and shape are highly heterogeneous in space and time. This problem is expected to be especially severe for deformable registration algorithms. In this paper, we analyze the effect of registration choice on inferences regarding the association between detailed lesion localization and adverse health effects in MS.

In particular, we focus on five commonly used algorithms implemented in four software platforms: the Advanced Normalization Tools (ANTS) described by [12], FMRIB Software Library (FSL) (see [13] for a general overview of FSL), Medical Image Processing Analysis and Visualization (MIPAV) (http://mipav.cit.nih.gov) and Statistical Parametric Mapping (SPM) (http://www.fil.ion.ucl.ac.uk/spm/). Our main conclusion is that the choice of the algorithm greatly impacts results of statistical parametric mapping.

We also extend current methods for statistical analysis in two ways: 1) by proposing an approach to account for disease duration and local lesion volume; and 2) by extending the voxel-wise regression approach by introducing a metric that measures the distance between the patient-specific lesion mask and the population average prevalence map. By using several voxel-wise regression models that contain hierarchical adjustment for confounders, we assess the effects of the choice of registration method on results.

## Materials and Methods

### Patients

The study consists of 98 patients in various stages of MS. The Johns Hopkins Medicine IRB acknowledged that the collection and analysis of data presented in this manuscript qualifies as exempt research under Department of Health and Human Services regulations. MRI and clinical data were previously collected as part of IRB approved research studies with written consent provided by participants. The identifiable MRI and clinical information accessed by the principal investigator were recorded in such a manner that subjects cannot be identified, directly or through identifiers linked to the subjects (e.g., no codes or links were retained to allow re-identification of individuals).

**Figure 1:**
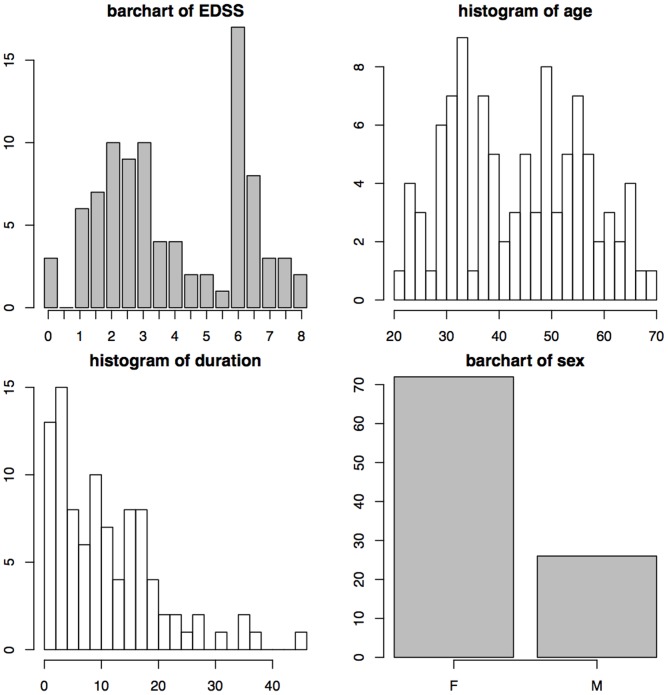
Histograms of EDSS scores, age (in years), sex, and duration from disease onset (in years) for the 98 patients in the study.

Histograms of EDSS scores, age, sex, and disease duration are shown in Figure 1, and descriptive statistics of age, disease duration and EDSS scores are shown in Table 1. The EDSS scores indicate high heterogeneity of disease burden, with a range between 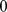 (normal neurological exam) to 8 (can be out of bed for a part of the day, but mostly restricted to bed or wheelchair, retains self-care functions). The distribution of age covers the range of values between 20 and 70 years of age somewhat uniformly with more subjects in the 30 to 40 years of age and 50–60 years of age ranges. Women comprise 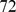
% of the dataset, which roughly reflects the relative sex distribution in the population. Finally, the distribution of duration from first symptom onset indicates that most patients have been experiencing symptoms for at least 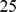 years prior to the scanning session.

**Table 1:** Descriptive statistics of the demographic information on the patients.

	min	median	mean	max	SD	NAs
EDSS	0	3.5	3.9	8	2.2	7
age	21.4	43.6	43.5	68.5	12.5	0
duration	0	9	11.4	45	9.2	3

### Imaging data

T1-weighted, T2-weighted, fluid-attenuated inversion recovery (FLAIR) and proton density (PD) volumes were acquired for all subjects. T1-MPRAGE images (repetition time (TR) = 10 ms; echo time (TE) = 6 ms; flip angle (FA) = 8; inversion time (TI) = 835 ms, 1.1 mm isotropic resolution), 2D T2-weighted pre-contrast FLAIR images (TR = 11000 ms; TE = 68 ms; TI = 2800 ms; in-plane resolution  = 0.83 mm
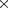
0.83 mm; slice thickness  = 2.2 mm), T2-weighted images and PD images (TR  = 4200 ms; TE  = 12 ms; in-plane resolution  = 0.83 mm
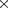
0.83 mm; slice thickness  = 2.2 mm) were acquired at the Kennedy Krieger Institute, affiliated with the Johns Hopkins University, on a Philips 3 tesla Achieva scanner (Philips Medical Systems, Best, The Netherlands).

### Lesion Segmentation

The T1-weighted images were rigidly aligned as described in the next section to the Montreal Neurological Institute (MNI) template and were used for segmentation of the lesions along with the coregistered FLAIR images. White matter lesions for all subjects were hand-segmented by a technician with over 10 years of experience in delineating MS lesions. For each image, the technician marked the lesion contours using a slice-by-slice approach. The hand-segmented lesion masks were used in the following analyses. While obtaining technician-drawn lesions is extremely time-intensive, these segmentations are considered the gold standard. In future studies that do not have hand-segmentations, we may consider automatic cross-sectional [14] or longitudinal [15] segmentation algorithms using the data from all modalities.

### Registration Algorithms

We applied five different registration algorithms to transform the data into template space. The first registration algorithm was a rigid registration of all subjects to MNI space. All image processing for the rigid registration was performed in the Medical Image Processing, Analysis and Visualization (MIPAV) application [16] with Java Image Science Toolkit (JIST) [17]. We first applied the N3 inhomogeneity correction algorithm [18] and removed extracerebral voxels using SPECTRE, a skull-stripping procedure [19]. The T1-weighted images for each subject were then rigidly aligned (no scaling was performed) to the MNI standard space (1-mm^3^ voxel resolution). The resulting rigidly aligned volumes were used as inputs for the other four registration algorithms and for the manual lesion segmentation.

The second registration algorithm is labeled “ANTS affine” and consisted of applying the affine registration implemented in the ANTS package [12] to the images obtained by rigid registration. Mutual information was specified as the similarity metric with a 4-bin square joint histogram and the weight of 1, and a Gaussian regularization term with sigma of 3.

The third registration algorithm is labeled “ANTS diffeo”, and consisted of applying the SyN[0.25] diffeomorphic model for transformation via the cross correlation similarity metric including a Gaussian regularizer with sigma of 1 with an 8-bin square joint histogram and the weight of 1 in the ANTS software [12] to the images obtained by rigid registration. In both the affine and diffeomorphic registration models performed via the ANTS software, the hand-segmented mask of the lesions was provided to focus the optimization algorithm on the area of the brain outside of the lesion mask.

The fourth registration is labeled “FSL nonlinear”, and consisted of applying the nonlinear registration tool in FSL, FNIRT [13], to the images obtained by rigid registration. Just as with ANTS, we provided the lesion masks in the registration algorithm. Default parameter settings were used for FNIRT while subsampling at levels of 4, 4, 2 and 1. The FNIRT tool in FSL provides only the nonlinear transformation of the images assuming that the images have already been linearly aligned. Hence, we first applied the FLIRT tool for affine registration of images into MNI space before proceeding to FNIRT registration.

The fifth registration algorithm labeled “DARTEL” consists of applying the Diffeomorphic Anatomical Registration Through Exponential Lie algebra (DARTEL) toolbox in SPM [20]. The T1-weighted images were segmented into six tissue types. Both white matter and gray matter images were used to construct an average group template and subject-specific flow-fields which were then used to register the lesion masks into the MNI space using a final affine transformation. Default settings were used for the variables in DARTEL with two exceptions during normalization to MNI space: we decreased the kernel size for the Gaussian filter applied to the data to 1-mm and specified 1-mm isotropic voxel size for our output images to match the resolution of the output images of the other registration methods. After directly applying DARTEL to the rigidly registered images we found that the registration quality was very poor. To alleviate the problem, we filled in the lesions with the average normal appearing white matter (NAWM) intensities before applying DARTEL. In other words, for each subject, the average NAWM intensity was assigned as the intensity of all voxels in each lesion [21]. LesionTOADS, an open source lesion segmentation software ([22], [23]), was used for segmenting the NAWM for the 98 subjects in the study.

The images with lesions filled with the average NAWM were used as inputs for “DARTEL”, whereas the inputs for “ANTS affine”, “ANTS differo”, and “FSL nonlinear” were the images with lesion voxels masked out as background. For the last three algorithms masking out the lesions is the recommended for preprocessing the data. To compare the registration results of all four methods for the exact same dataset with average NAWM assigned as the intensity of each voxel in each lesion, we used “ANTS affine”, “ANTS differo”, and “FSL nonlinear” to register these data as well as the data with masked lesions.

**Figure 2:**
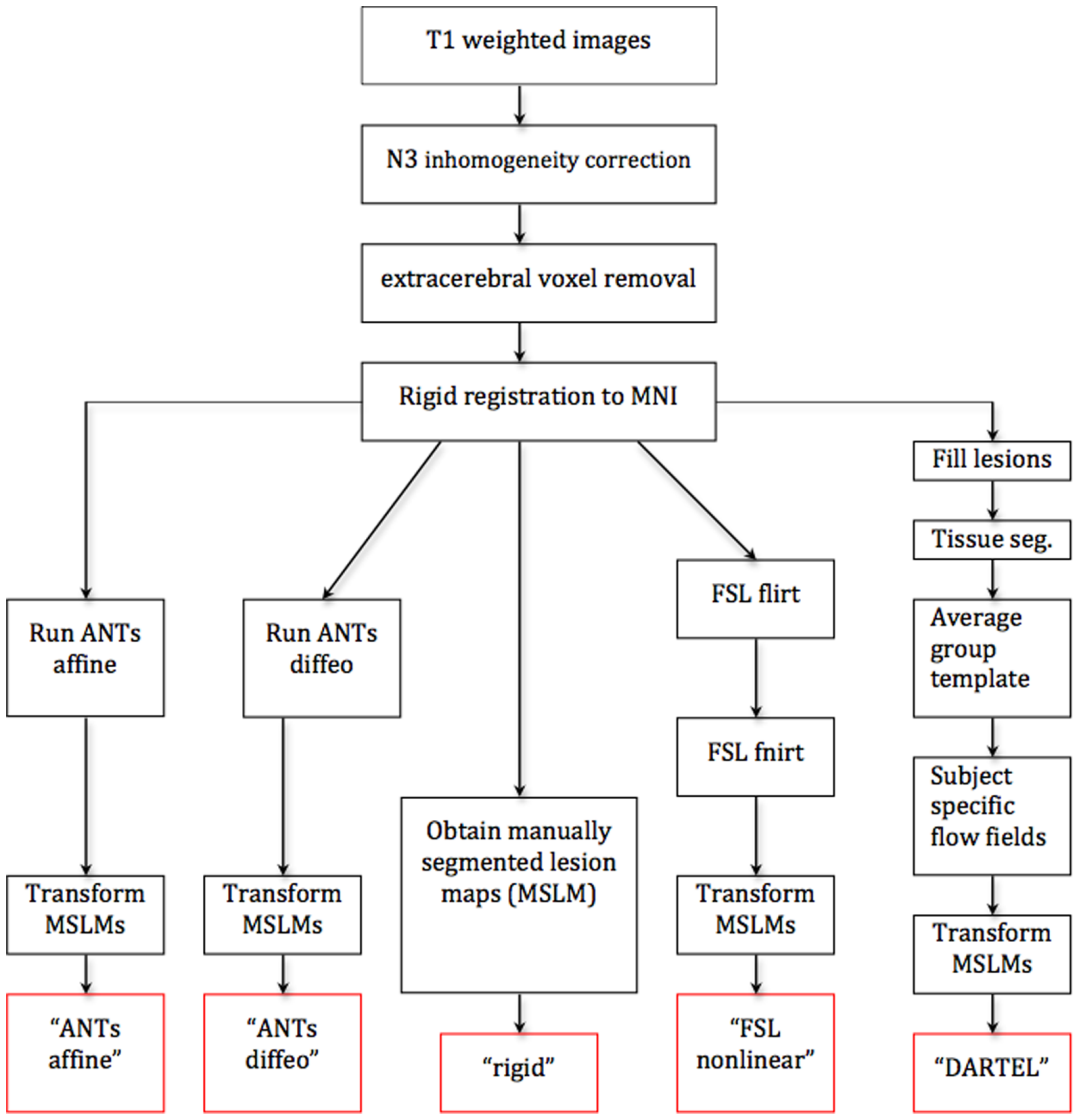
Review of the steps behind the five algorithms.

**Figure 3:**
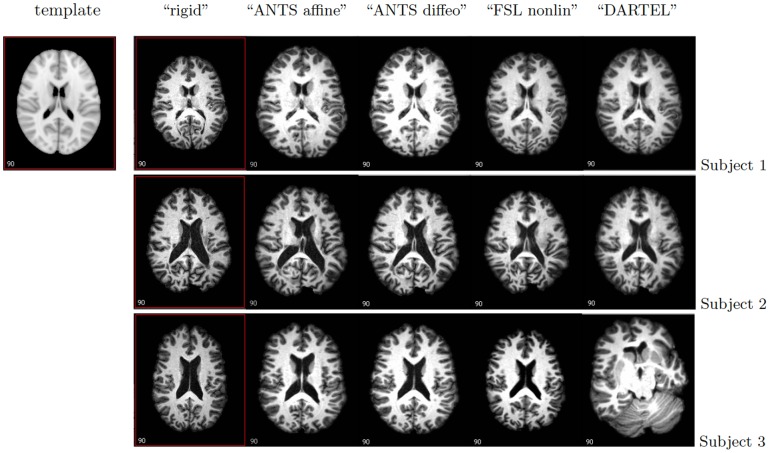
One slice from five different registration methods for three subjects (one subject on each row). The MNI template brain is shown on the first row (left).

A visual representation of the five registration procedures is presented in Figure 2. The registered images for three subjects are shown in Figure 3 with the corresponding labels. We chose two subjects with enlarged ventricles and one subject with small ventricles. Images indicate that gross brain features remain relatively unchanged, though important differences that can impact results in subsequent statistical parametric mapping can easily be noticed. A visual inspection of the differences between the algorithms is discussed in the next section.

### Assessing Registration

Quantifying the quality of spatial registration algorithms is a daunting task: 1) there is no gold standard for assessing registration results; 2) current approaches are based largely on qualitative comparative assessments of the original and registered images; and 3) there is a lack of systematic analyses of implications of registration in populations of brain images, especially when the subjects in the population exhibit obvious pathologies. Landmark-based measures have been used in the literature to compare the effectiveness of registration algorithms [24]. The general consensus favors nonlinear registration algorithms such as the SPM DARTEL method used in this manuscript [25].

Here we propose an approach to assessing the quality of registration in the context of MS brain imaging. We start by performing a visual inspection of the results and notice that the relative effectiveness of the five registration approaches varies as individual anatomy deviates from the template. When the ventricles are only slightly larger than normal as for Subject 1 in Figure 3, ANTS affine, ANTS diffeo, FSL Nonlinear as well as SPM DARTEL transform the image into the template space better than a simple rigid transform. However, when ventricles are very large, as is typical in subjects with MS, the quality of the registration becomes harder to assess and interpret. This happens because brains that have fundamentally different pathologies and shapes are being forced onto the same template. While this is perhaps not surprising, it should be of great concern to researchers who apply these registration methods to diseased populations, especially because it is hard to anticipate when registration will work well or fail. For example, Subject 2 in Figure 3 has both enlarged ventricles and a high total lesion volume, and both FSL Nonlinear and SPM DARTEL seem to perform better than the other registration methods. For Subject 3 in Figure 3, who has enlarged ventricles and low lesion volume, DARTEL performs very poorly.

In order to develop a metric to evaluate registration accuracy, we note that the visible lesions segmented by the technician are all in white matter. Thus, it makes sense to expect the lesions to remain in the white matter of the template after registration is performed. Hence, we propose the following goodness-of-lesion-spatial-registration (GLSR) metric at the subject level: the proportion of lesion voxels in the original unregistered image that do not appear in the white matter of template space after registration. By this definition, a low GLSR indicates that white matter lesions correctly remain in white matter, without measuring the relative position of the lesions with respect to various landmarks. While this is a partial solution, the idea can easily be extended to registration in general: manually identify landmark areas in the native space of the images, such as gray matter, regions of interest (ROI) such as a brain tumor or the relative spatial location of an ROI from a predefined landmark, and obtain the proportion of voxels in the template, after registration, that are misplaced. While a more complete solution is desirable, it is beyond the scope of this manuscript. Instead, we focus on the GLSR metric henceforth.

### Prediction of EDSS Based on Lesion Localization

For each registration algorithm, we evaluate the association between lesion localization and disease severity as measured by the EDSS score. At every voxel in the template space, we ran a linear regression model of the type



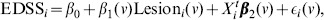
(1)where 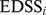 is the EDSS score for subject 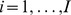 with 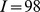, 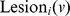 is a binary covariate that indicates whether subject 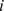 has a lesion at voxel 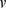, 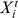 is a vector of subject-specific confounders, and 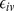 are treated as independent homoscedastic errors. We limit our analysis to voxel locations where more than 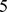 patients have lesions. The vector of confounders, 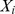, contains up to 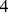 subject-specific confounders including age and sex. A third interesting confounder is disease duration, as some areas in the brain may accumulate lesions over time without having a strong impact on the EDSS. Thus, not accounting for disease duration would naturally identify strong associations in high lesion density areas simply because MS subjects' health tends to deteriorate over time. A fourth confounder was designed to account for potential association between EDSS score and lesion locations that are less common across subjects. More specifically, for each subject, we calculated a distance from the subject-specific lesion mask to the sample lesion mask; this was done separately for each registration. Here the sample lesion mask is defined as the collection of voxel locations in the template space where more than 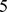 subjects had a lesion. We define as 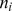 the number of voxels where subject 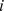 has a lesion and there are no more than 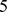 other subjects (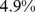 of the study population) who have a lesion at the same voxel. If the total lesion volume (TLV) of subject 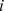, denoted by 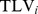, is the total number of voxels in the lesion mask of subject 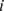 then we define the location-discrepancy index (LDI) as



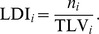



The 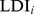 measure takes values between 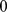 and 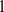, with a large value indicating a more atypical lesion pattern for subject 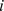. Subjects with very large values of 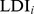 may be viewed as outliers in the sense of their lesion structure. 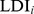 allows us to model the degree of departure from the average lesion distribution. TLV can also be considered as a confounder in the model. Indeed, a larger TLV may be associated with a longer time since disease onset.

## Results

### Histograms of Lesions in the Brain

**Figure 4:**
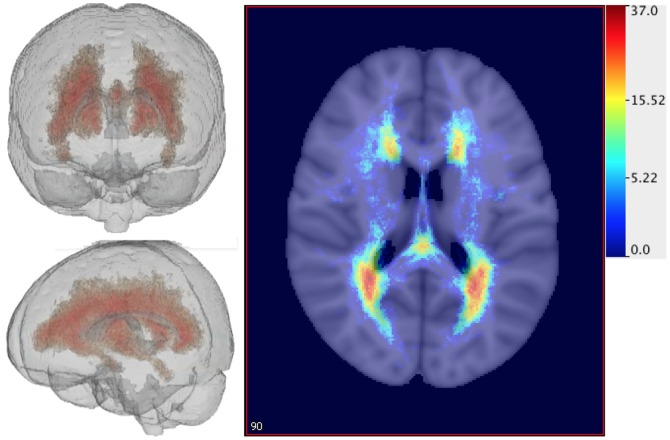
The histogram of brain lesions for 98 patients based on a rigid registration of the images to a template brain indicating the number of patients out of 98 having lesions at each voxel.

To visualize the localization of lesions, we first combined information from lesion masks of 98 patients with MS. As mentioned above, lesions were manually segmented in the rigidly registered space. Using the resulting lesion masks we obtained the 3 dimensional histogram of the lesion localization for our study population. More precisely, for every voxel location we calculated the proportion of subjects who have a lesion at that particular voxel. The resulting image based on the rigid transformation of the data is shown in Figure 4. Here the voxels colored in red correspond to a higher proportion of patients with lesions at that voxel whereas light blue color indicates voxels with a smaller proportion of patients having lesions at that voxel. The histogram indicates that lesions are fairly widely distributed in the brain, with a higher concentration around ventricles.

**Figure 5:**
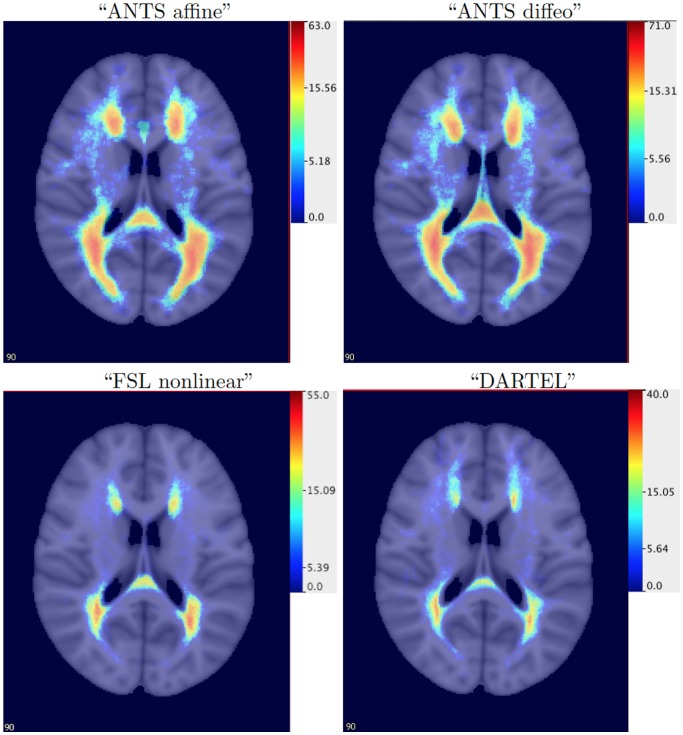
The lesion histograms for 98 patients (showing the number of patients out of 98 having lesions at each voxel) based on “ANTS affine” (top left), “ANTS diffeo” (top right), “FSL nonlinear” (bottom left) and “DARTEL” (bottom right) spatial registration algorithms. Red: voxels where more patients have lesions; Blue and light blue: voxels where fewer patients have lesions.

Similar histograms were obtained for “ANTS affine”, “ANTS diffeo”, “FSL nonlinear” and “DARTEL”; see Figure 5. The “ANTS affine” and “ANTS diffeo” registrations appear to have shifted many of the lesions away from the ventricles of the template space; compare the results for “rigid” displayed in Figure 4 with the first row in Figure 5. The “FSL nonlinear” and “DARTEL” registration methods lead to results that seem to be qualitatively different from the first two registrations. The 3D histograms for “FSL nonlinear” and “DARTEL” indicate a smaller number of lesions and a different spread pattern of lesions, which suggests that the registration procedures can either reduce or enlarge the size of the lesions in the same template space. Recall that the hand-segmented lesion masks are provided for the “ANTS affine”, “ANTS diffeo” and “FSL nonlinear”. The 3D histogram for “DARTEL” indicates that lesions are registered outside of the ventricles in the template image, however, the localization of lesions seems to be more spread out as compared with the rigid registration map especially in anterior regions of the brain.

The differences between spatial registration techniques observed using a simple visualization tool such as the 3D histogram are striking. These differences may directly affect the reliability and interpretation of statistical parametric mapping. The main question of interest in this paper is whether and to what extent are the results of studies of association between lesion locations and disease severity affected by the registration algorithm or biologically irrelevant factors.

### Assessing the Goodness-of-Lesion-Spatial-Registration

While the previous visualization approaches provide insights into the data and the distortions induced by spatial registration, the quantification of observed results remains elusive. Thus, we focus on quantification of the Goodness-of-Lesion-Spatial-Registration (GLSR). At the subject level, we define GLSR to be the proportion of voxels that are in lesions in the images in native space, but are not in the white matter after registration. FAST in FSL was used to segment the white matter in the template brain. Thus, GLSR is a measure that depends both on the subject and on the registration approach.

**Figure 6:**
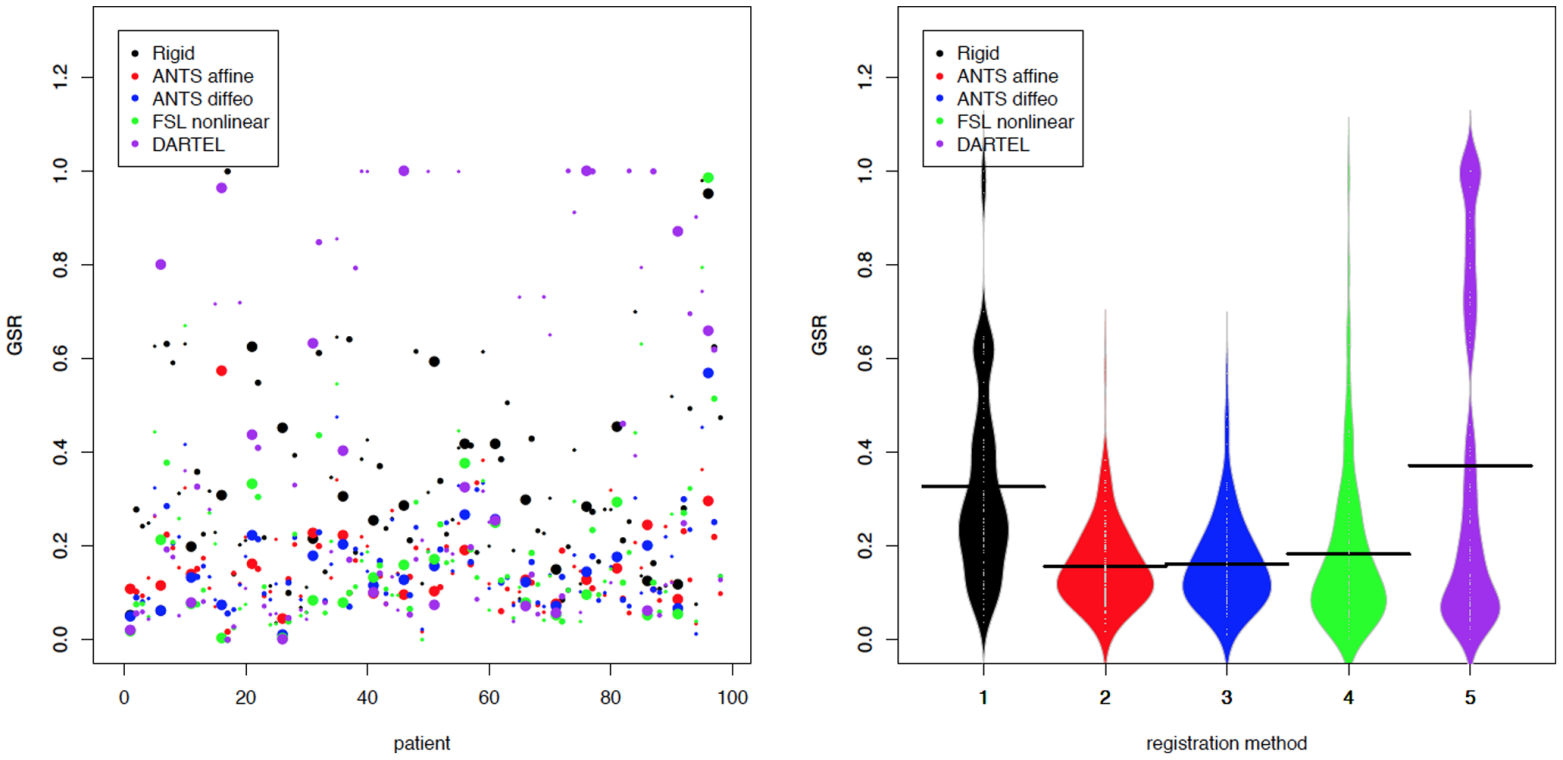
GLSR: the proportion of lesion voxels for each registration algorithm that are not in the white matter in the template space. Each dot (left) is the GLSR for one subject for a particular registration algorithm: “rigid” (black), “ANTS affine” (red), “ANTS diffeo” (blue), “FSL nonlinear” (green) and “DARTEL” (purple). Higher and more variable across subjects is worse. The size of the dots is proportional to the TLV for each patient. Larger dots correspond to higher TLV. The beanplots (right) show the distribution of GLSR for each registration algorithm.

Figure 6 displays GLSR for all subjects and each registration procedure: black for “rigid”, red for “ANTS affine”, blue for “ANTS diffeo”, green for “FSL nonlinear” and purple for “DARTEL”. Table 2 shows the mean, standard deviation (SD), average ranking of each method across subjects and the mean squared error (MSE) of GLSR across subjects for each of the registration algorithms. The MSE is defined as

**Table 2:** Properties of the GLSR statistic computed for each of the registration algorithms (higher is worse).

	“rigid”	“ANTS affine”	“ANTS diffeo”	“FSL nonlinear”	“DARTEL”
mean	0.32	0.16 (0.17)	0.16 (0.16)	0.18 (0.25)	0.37
median	0.27	0.13 (0.15)	0.14 (0.14)	0.12 (0.21)	0.19
SD	0.206	0.088 (0.12)	0.099 (0.11)	0.173 (0.14)	0.349
MSE	0.148	0.032	0.035	0.063	0.259
mean rank	4.23	2.55	2.62	2.44	3.14

The values in the brackets show the corresponding statistic computed while filling the lesions with average NAWM before registration.



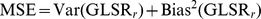
where 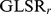 is the statistic computed for registration algorithm 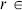 (“rigid”, “ANTS affine”, “ANTS diffeo”, “FSL nonlinear”, “DARTEL”). The 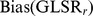 of the estimator is the mean bias of the 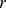
th algorithm GLSR with respect to the truth, which, in this case, is GLSR = 0 (white matter lesions should be registered to white matter).

First, all methods exhibit some bias and variance, though DARTEL is by far the most variable. In fact, DARTEL, exhibits good performance in a large subset of subjects and fails completely in others. As expected, on average “rigid” performs badly, though it exhibits low variability around bad values of GLSR across subjects. We conclude that “rigid” performs consistently, but poorly. This is probably due to the fact that the shape of the original brain is preserved while the white-matter overlap is poor. Surprisingly, we found high variability in the performance of “DARTEL” in registering the lesions to the white matter of the template space correctly in terms of GLSR with a mean GLSR of 0.37 and standard deviation of 0.349. In fact for 6 subjects out of 98 the total white matter lesion volume after registration was equal to zero. “ANTS affine”, “ANTS diffeo” and “FSL nonlinear” registration procedures shift lesions to overlap better with white matter, though “ANTS diffeo” seems to perform the best on average, at least in terms of GLSR. Indeed, its average GLSR is 0.16 with a standard deviation of 0.099. “ANTS affine” performs similar to “ANTS diffeo” and “FSL nonlinear” with mean GLSR of 0.18 and standard deviation of 0.173. However, in terms of median GLSR, “FSL nonlinear” outperforms all other methods with a median GLSR of 0.12. In addition, “FSL nonlinear” is ranked as the better algorithm on average in terms of minimizing GLSR. The complete distribution of GLSR for each registration algorithm is displayed in Figure 6. We conclude that the performance of all algorithms is worrying, with especially unpredictable behavior for “DARTEL”, which performs very badly in a large proportion of subjects (e.g. 10% of the subjects have GLSR larger than 0.9).

The resulting GLSR values for “ANTS affine” (mean GLSR of 0.17, median GLSR of 0.15, and standard deviation of 0.12), “ANTS diffeo” (mean GLSR of 0.16, median GLSR of 0.14, and standard deviation of 0.11), and “FSL nonlinear” (mean GLSR of 0.25, median GLSR of 0.21, and standard deviation of 0.14) when the lesion voxels of the input images were assigned the average NAWM intensity showed that masking the lesions as background is indeed better in terms of GLSR, especially for “FSL nonlinear”.

Admittedly, when registering individual images, one may select the parameters to better register the individual brains to the template space for each of the registration algorithms. However, the purpose of this paper is to show the performance of the methods for a population of patients. While we tried different settings of parameters for three of the registration algorithms, “ANTS diffeo”, “ANTS affine” and “FSL nonlinear”, we chose parameters for the population and did not change them for individual images. As mentioned above, the parameters for “DARTEL” were used as specified by the software.

Interestingly, we found some differences in total lesion volume for each subject depending on the registration algorithm. The average total lesion volumes for “rigid”, “FSL nonlinear” and “DARTEL” were similar, however the average total lesion volume for “ANTS affine” and “ANTS diffeo” was 3 times that of the average total lesion volume for “rigid”. As research often focuses on much smaller signals, these differences raise questions about the reliability and reproducibility of findings especially in the context of voxel-wise analysis of the data where the sheer size of the lesions may dominate the results.

While lower GLSR numbers can indicate better registration, the measure is far from perfect. Indeed, a low GLSR indicates that white matter lesions correctly remain in white matter, without measuring the relative position of the lesions with respect to various landmarks. Indeed, while “ANTS diffeo” performs well at keeping lesions in the white matter it may actually do so at the price of “over-smearing” the image and providing visual representations of lesions in areas of the white matter that are too far from the ventricles. In addition, “ANTS affine” and “ANTS diffeo” methods seem to enlarge lesions, which results in a larger lesion volume in the template space compared to the size of those lesions in native space. Currently, we are unaware of any existent quantitative method for assessing this problem.

### Prediction of EDSS Based on Lesion Localization

We run several versions of model (1) starting with the simplest case and then building the model by incorporating different choices of potential confounders to investigate their effects on the statistically significant association between lesion locations and EDSS. More precisely, the five models are:


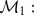
 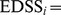 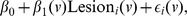



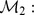
 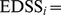 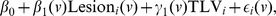



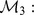
 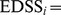 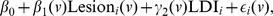



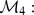
 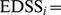 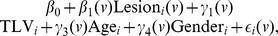



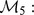
 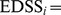 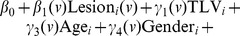 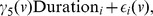


**Figure 7:**
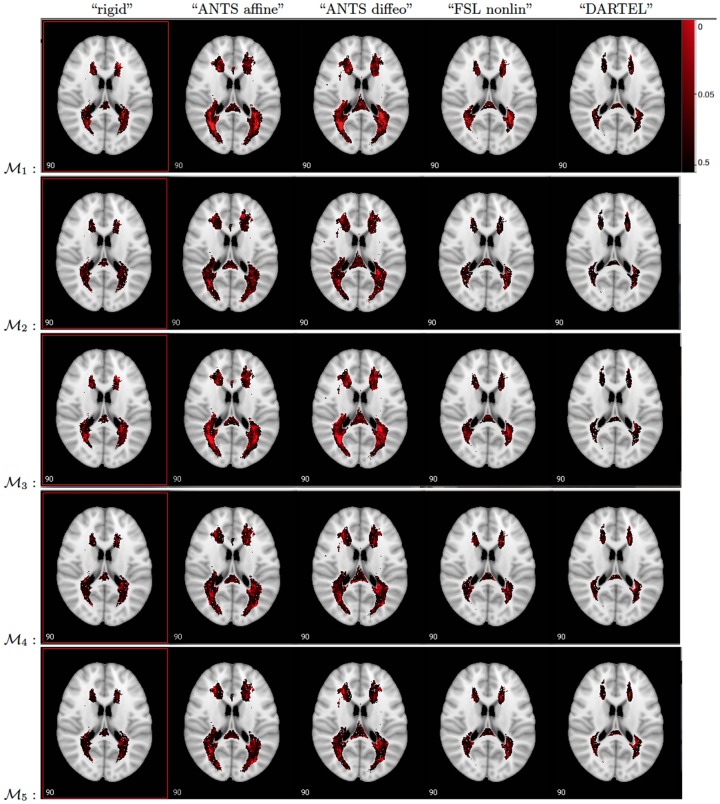
P-values (uncorrected) for testing 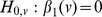 using models 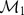
–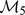. From left to right: spatial registrations “rigid”, “ANTS affine”, “ANTS diffeo”, “FSL nonlinear”, and “DARTEL”. Bright red: p-values close to 0 to black: p-values close to 1. The p-value maps are overlaid on a grayscale template.

The p-values of voxel-wise regression of EDSS scores on lesion incidence vectors corresponding to the test 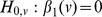 are shown in Figure 7. Each row in Figure 7 corresponds to one of the models above. In the first model - 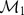 in the top row, the p-values based on the “ANTS affine” and “ANTS diffeo” registration are smaller with a clear spatial distribution of p-values smaller than 0.05 near the ventricles. Patterns of p-values that are smaller than 0.05 are far less clear for the “rigid”, “FSL nonlinear” and “DARTEL” registration approaches. In all cases, the areas highlighted in bright red display p-values of less than 0.05, however none survive Bonferroni correction.

The first model indicates that if a patient has a lesion in a voxel near the ventricles, their disease severity score may be higher than if they do not have a lesion. An interesting question is whether the lower p-values in the last analysis are indicative of structural association between lesion locations and EDSS or whether that is merely a factor that indirectly shows how long the patient has been affected by MS. In other words, the relationship between lesion location and EDSS may be confounded by the total lesion volume (TLV) measures in mm^3^. We first note that if we compute a registration specific TLV the numbers vary depending on the registration algorithm. This can be observed in the lesion histograms in Figure 5. Using a simple linear regression of EDSS on the registration specific TLV for each of the 4 registration algorithms, we find that the TLV is a significant predictor of EDSS based on the first four registration algorithms with the following coefficients and p-values: “ANTS affine” 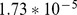 (0.0166), “ANTS diffeo” 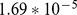 (0.0188), “FSL nonlinear” 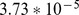 (0.0178) and “rigid” 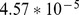 (0.0083). The analysis indicates that as the TLV in the patients increases, the EDSS score increases, as expected. However, for “DARTEL”, the p-value was found to be 0.074 indicating that TLV is not a significant predictor of EDSS. These results are based on four simple linear regression models where EDSS is the dependent variable and the TLV is the predictor and no multiple comparisons correction is used.

In the second model 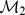, we used TLV as a second variable in the regression model along with the indicator variable of lesion prevalence. The second row of Figure 7 shows the resulting p-values for the 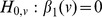. The p-values for testing 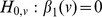 are now larger. This suggests that for subjects with the same total lesion volume, the statistical association between the specific location of the lesion and the EDSS is not as strong as that estimated by 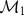.

In the model 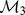, we incorporated the new measure of lesion discrepancy index (LDI) along with the indicator variable of lesion incidence. The p-value maps for 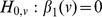 displayed in Figure 7 indicate smaller p-values. A simple linear regression of the EDSS score on the LDI for each of the registration algorithms results in p-values as follows: 0.47 - “rigid”, 0.09 - “ANTS affine”, 0.12 - “ANTS diffeo”, 0.06 - “FSL nonlinear” and 0.05 - “DARTEL”. These results are consistent with those from model 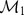 and indicate insufficient statistical evidence of association between the degree of discrepancy of the subject-specific lesion pattern from the average distribution of lesions and EDSS score.

The effects of the demographic variables such as age, duration of the disease, and gender of the patients are explored in models 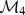 and 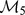. In model 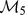, age is a significant predictor of EDSS after accounting for gender and lesion incidence. Many of the p-values are significant for testing 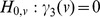 (localized age effect) after Bonferroni correction, while gender and duration of disease are not significant predictors of EDSS with high p-values for tests 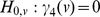 and 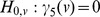. We observe similar results for model 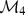 where age is a significant predictor of EDSS.

**Figure 8:**
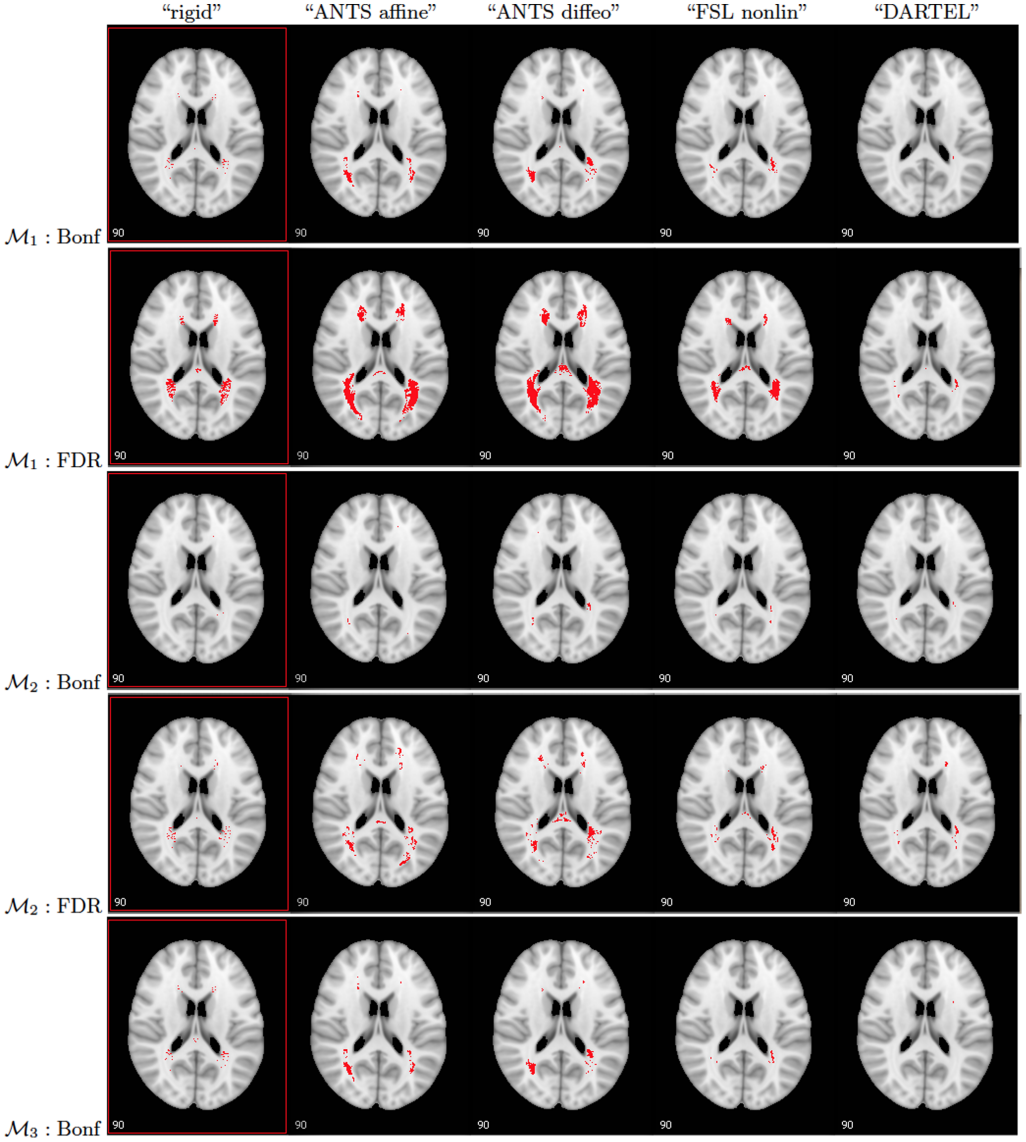
P-values for testing 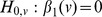 using models 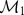
–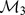 after applying Bonferroni and FDR corrections. From left to right: spatial registrations “rigid”, “ANTS affine”, “ANTS diffeo”, “FSL nonlinear”, and “DARTEL”. Red: small p-values (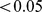).

As noted above, none of the p-values for testing 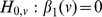 (localization effect on EDSS) obtained from models 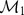
–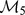 survived a Bonferroni correction for multiple comparisons. A major difference between our findings and those of [3] is that they had more than 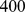 subjects, whereas we only had 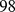. Thus, we re-assessed our results by simulating the scenario of having 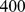 subjects instead of 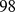 and re-calculating p-values. This can be approximated simply by dividing our standard errors by 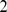. The newly “calculated" p-values were then corrected for multiple comparisons via two methods: the Bonferroni correction and False Discovery Rate (FDR). The thresholded maps of the resulting p-values at the 0.05 level are shown in Figure 8.

If Bonferroni correction is used, then 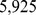 lesion voxels out of 52,821 tests performed simultaneously were found to have a slope statistically different from zero using “ANTS affine”, 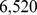 out of 54,614 using “ANTS diffeo”, 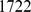 out of 19,497 using “FSL nonlinear”, 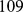 out of 10,119 using “rigid”, and 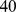 out of 11,918 using “DARTEL” registration and model 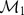. The results are drastically different when using FDR for multiplicity correction. For instance, the number of significant slopes when “ANTS affine” is used for registration in Model 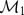 increases to 38,196 as compared with the 5,925 in the case of Bonferroni correction. This would lead to a different conclusion in identifying regions of the brain where the presence of lesions could be assumed to be correlated with the EDSS score. Furthermore, when including TLV in the model, the number of lesion voxels that were found to be significantly associated with the EDSS score was reduced dramatically to 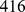 based on “ANTS affine”, 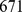 based on “ANTS diffeo”, 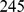 based on “FSL nonlinear” and 128 based on “DARTEL” in model 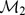. The results from models 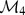 and 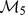 were similar to the results from model 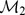.

After correcting for the effect of the proposed LDI measure in model 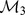, the number of slopes that are statistically significant based on “ANTS affine” registration was 6,901, based on “ANTS diffeo” - 7,253, based on “FSL nonlinear” - 566 and based on “DARTEL” - 23 which suggests that for the patients with similar LDI measures, the probability of having a lesion in many areas of the brain has a significant effect on their EDSS score, however, this conclusion does not hold for most voxels if “FSL nonlinear” or “DARTEL” registration methods were used.

**Figure 9:**
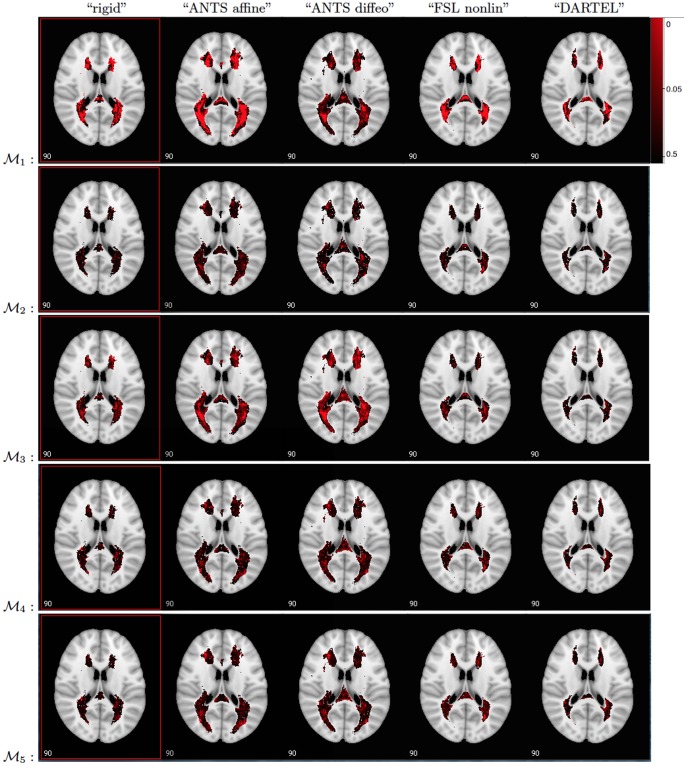
P-values (uncorrected) for testing 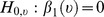 using ordinal regression models with independent variables as in models 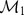
–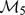. From left to right: spatial registrations “rigid”, “ANTS affine”, “ANTS diffeo”, “FSL nonlinear”, and “DARTEL”. Bright red: p-values close to 0 to black: p-values close to 1. The p-value maps are overlaid on a grayscale template.

So far, the EDSS score was treated as a continuous variable to allow for a direct comparison with published studies. However, EDSS is intrinsically an ordinal variable with 20 categories ranging from 0 to 10. To account for the ordinal nature of the data, we have also conducted analyses using the observed data where subjects were divided into three categories: EDSS<4, EDSS between 4 and 5.5, and EDSS> = 6. An ordinal regression model [26] was then used to study the association between lesion localization and EDSS score, treated as an ordinal variable with three levels. The resulting p-value maps are presented in Figure 9. The results are qualitatively similar to those obtained when EDSS was treated as a continuous variable.

It is worth mentioning that family-wise error rate (FWER) and false discovery rate (FDR) are intrinsically different quantities and there is no reason to expect similar results. Indeed, the Bonferonni correction ensures that, irrespective of the number of tests and correlation between them, the FWER is at most equal to a pre-defined upper limit, say 0.05. In contrast, the FDR ensures that the proportion of falsely null hypotheses that are rejected is controlled below a certain rate, say 0.05. Both FWER and FDR are defined for independent tests, though the Bonferonni correction provides stricter controls (higher thresholds for declaring a positive result).

## Discussion

We began this project as a study of association between lesion localization in multiple sclerosis and EDSS. However, during the course of the project our focus shifted, as we encountered several methodological hurdles. The analyses presented in this paper were crafted to illustrate the influence of image registration variation and the effect of other confounding variables on the association between lesion localization and MS severity as measured by the EDSS score. One of the most important findings of this paper is that spatial registration algorithms that have been developed in the context of registration of healthy brains provide mediocre results for brains of subjects with multiple sclerosis pathology. In addition, the effectiveness of different spatial registration algorithms can lead to fundamentally different conclusions when one is quantifying the association between lesion localization and health outcomes. Consequently, much more in-depth analyses are needed to quantify goodness of registration and comparison across platforms and diseases. We also demonstrated that after accounting for multiple testing and controlling for total lesion volume, the strength of the association between localization and EDSS score is much weaker. Finally, in our sample, particular patterns of lesions as measured by LDI do not seem to be significantly associated with EDSS score.

In an attempt to quantify the registration accuracy we proposed a new Goodness-of-Lesion-Spatial-Registration measure as the proportion of lesion voxels in the transformed image that are not in white matter, which is essentially a measure of a combination of tissue classification and spatial registration. When comparing the five different registration algorithms via GLSR, we find that the ANTS and FSL perform better than SPM or rigid registration. When comparing the number of lesion voxels in ANTS registered images with FSL or SPM registered images, we found that the TLV for “ANTS affine” and “ANTS diffeo” are much higher than the TLV for “FSL nonlinear”, “rigid” and “DARTEL”. There are several other aspects of the registration accuracy that can be considered, such as whether the lesion volumes are preserved after the transformation or if the lesions have the correct placement in the template space in reference to other brain regions, etc.

The segmentation/registration of images using SPM was most troubling for some of the subjects in the study. As discussed above, we used the parameter settings recommended by SPM8 (with 2 exceptions: we decreased the kernel size for the Gaussian filter applied to the data to 1-mm and specified 1-mm isotropic voxels for our output images to match the resolution of the output images of the other registration methods). We first used the rigidly registered images as inputs for SPM's segmentation procedure and the resulting gray and white matter images as inputs for DARTEL, and found that the performance of the method was much worse (results not shown). We assumed that since we did not exclude the white matter lesions from the rigidly registered images as we did with ANTS and FSL implementations, these areas may have been mislabeled as gray matter by SPM [6]. To mitigate the issue, we proceeded by filling in the lesions with average normal appearing white matter based on the white matter segmentations we obtained from FSL FAST. When using the new images as inputs for SPM's segmentation procedure and the resulting gray and white matter images as inputs for DARTEL, the results improved for some of the subjects, though overall the performance of the algorithm remained dismal for a number of subjects. Interestingly, most of the subjects who continued to have very high GLSR values had low lesion loads. After further investigation of the SPM segmentation results, we have found that the SPM tissue segmentation of these images was poor; this could be a possible explanation for some of the high GLSR values.

In addition, DARTEL works by iteratively estimating the deformations that match subject-specific tissue class images to a template and then using the latest deformation estimates to update the template [27], [28]. Because the final template used for registration depends on all subject-specific tissue segmentations, inclusion of poorly classified gray and white matter images from several subjects as inputs to DARTEL could reduce the effectiveness of DARTEL to register images even from subjects whose gray and white matter tissue had been adequately classified. Hence, it may be reasonable to exclude the subjects with high GLSR values from the study to improve the registration accuracy for the population.

One decision that might have contributed to the worse-than-expected performance of all of the algorithms is that we used the rigidly registered images as inputs for all the algorithms whereas it may be reasonable to try and minimize the number of image interpolation steps and use the observed images in the native space as inputs for each registration algorithm. Nevertheless, some algorithms, such as FNIRT in FSL, require input images that have already undergone an affine registration. When we used the rigidly registered images in our first run of “FSL nonlinear” the resulting GLSR values were very high and the registration was poor. Hence, it is important to check the steps of the registration procedures as well as check the results by a metric such as GLSR before commencing the statistical analysis.

Another important point is that the healthy MNI template was used for registration. As a result the highly deformed brains are registered to the template that was constructed from typical brains. In some studies it may be reasonable to construct a study specific template for population-level analysis; however, in that case, comparisons between analyses may be impossible. An important open question is what types of features one might expect in a nonlinearly registered brain in terms of the deformation of lesions and other structures of the brain. This problem extends to healthy brains as well, since brain tissue is lost with age. In MS particularly, a significant amount of tissue may be lost as the disease progresses. Hence, when nonlinearly aligning an MS patient's scan to any template the algorithm is often trying to register the remaining tissue to the lost structure. There are no guidelines for the algorithm for performing this, nor can there be, since we may not know what tissue is lost in the MS patient's brain. The results can be different depending on the optimization routine used by the algorithm. This can adversely affect the analysis of the resulting images, especially in the case of voxelwise inference of associations.

Some studies described the relationship of lesion localization in MS as compared with other neurological disorders such as neuromyelitis optica spectrum disorder [29]. [30] and [31] commented on the relevance of lesion location in clinically isolated syndrome (CIS) patients when predicting the short-term conversion to MS, whereas [32] investigated the effect for up to 20 years after presentation of CIS in MS patients. [33] discussed the distribution of lesions in MS patients with fatigue. Even though the proposed lesion discrepancy index was not found to be significantly associated with the EDSS score, it may be useful in diagnosing MS and distinguishing MS from other causes of white matter lesions. A study of patients with white matter lesions caused by different neurological disorder will be of interest in finding associations of LDI with the type of disease that causes lesions in the white matter. In addition, patients with small vessel disease along with MS may have more randomly spread out white matter lesions in unexpected locations as well as MS patients with a very high lesion load which would result in a high LDI value for these patients. The association of LDI with other metrics of disease burden can be discussed for a study with more patients in various stages of the disease.

In summary, our results show that: 1) conclusions of studies of association between lesion localization and health outcomes can vary strongly with the spatial registration method; 2) spatial registration methods to a template perform relatively poorly with a quality of registration that can vary dramatically in the population; 3) adjusting for known and potential confounders is crucial for the purpose of building credible statistical results; 4) adjusting for multiplicity in the absence of strong prior hypothesis is necessary and strongly influences the interpretation of results; and 5) irrespective of how carefully the image predictor space is constructed, the choice of phenotype (e.g. EDSS score) can strongly influence discovery, especially when it is subject to substantial heterogeneity (lack of precision).

Our approach in this paper was to point out these problems, provide a list of pre-processing steps and modeling choices that have a large effect on the reports of analyses, and propose ways to identify, quantify, and understand these effects. To address the dependence of findings on registration algorithm, we propose to apply all registration algorithms, do a side-by-side comparison of results, and construct reasonable metrics for goodness of registration. Comparing these goodness of registration metrics across all subjects provides a general description of registration performance and can highlight cases that are particularly problematic for future analyses. In terms of the metric considered in this paper (proportion of white matter lesions that remain in the white matter of the template), all registration methods performed poorly. Thus, we have identified a strong need for better registration methods or a change towards analytic methods that do not require or are robust to registration to a template. Changing the template to one that is more suited for the study or using multiple templates may be useful, though it may also affect the interpretability and generalizability of results. Adjusting for confounders is another crucial element of the analysis, as we need to quantify the type of information that imaging provides above and beyond known predictors. For example, it is well known that MS disease progression is associated with increased total lesion volume. In addition, we showed that the association between EDSS and lesion location may be strongly confounded by several variables. For example, total lesion volume (TLV) was found to be highly associated with EDSS and to strongly reduce the strength of the association between lesion localization and EDSS. We also showed that there is a higher density of lesions close to the ventricles, which raises the question whether having a lesion close to the ventricle versus elsewhere in the brain is more strongly associated with adverse health effects. Once we corrected for disease duration we found that there is not enough evidence to support such a hypothesis. An analysis that ignored disease duration would thus provide a completely different picture of findings that would be publishable, but probably misleading.
